# A Perfumed Solution of Iodoform

**Published:** 1879-01

**Authors:** 


A Perfumed Solution of Iodoform.—The objection to the use of iodoform, on account of its unpleasant odor, says the *Medical and Surgical Reporter*, has led an English druggist to prepare a perfumed solution. This is prepared by shaking tincture of iodine with a fragment of fused potash until the odor is removed, and covering the odor of the iodoform produced by the addition of eau de cologne or lavender water. The author speaks of lint that has been dipped in this liquid, and afterward dried, as being a very good application to indolent sores.—*Richmond and Louisville Med. Jour.*

